# Communicated

**Published:** 1864-12

**Authors:** H. Webster Jones


### COMMUNICATED.

MESSRS. EDITORS:

The task of the medical reformer is always, like that of Sisyphus of old, an endless one. Hardly has he elaborated his idea, and brought it into public notice, when ignorance and prejudice combine to roll it back upon him. Even when the summit of success is reached in the general recognition of its usefulness, his strife is not to end amid the rivalry of conflicting and opposing views of individuals.

Not to affect the *role* of the professional philanthropist, may I offer to your readers some objections to the continued proclamation of the virtues of this or that method in the treatment of *prolapsus uteri*, whose success, if it be attained, is unnatural and abhorrent to our merest instincts, not to say enlightened reason; concluding with some brief statements which may incline an inquiring mind to a more careful examination of other means applicable to the vast majority of cases, and interfering in no way with the functional or organic perfectness of the patient.

To the American mind the idea of curing prolapse of the uterus by inducing the cohesion of vaginal walls is singularly repugnant. One readily conceives of the possibility of closing the vaginal outlet, thus to prevent the actual exit of a *procident* womb, but in practice the most distressing cases are those in which but slight prolapse is present; where, if it be true as our veterans in obstetric science assert, that the vaginal walls by themselves considered are in no wise the uterine support, this severe and unnatural operation would be superfluous. Indeed, since vaginal contractions are the most dreaded obstacles to the reposition and maintenance of a displaced organ, *artificially* induced by the actual cautery, they must not only fail in purpose, but absolutely preclude all further effort to relieve the injured and suffering patient. The attempt to excise a portion of the relaxed duplicatures of peritoneum, which constitute the true uterine and vaginal ligaments, thus to shorten them and support the womb, would seem more philosophical and scarely more barbarous.

The vagina is more properly to be esteemed an antagonizing force to the uterine ligaments, if the term force may be applied to so passive an organ. Its relaxation therefore can not, alone, predispose to uterine prolapse, though its contraction would most assuredly do so, and I apprehend that proper investigation would assign a very prominent place to this cause in displacements occurring after severe and tedious labors. I repeat, therefore, my conviction that Dr. Bird's operation, acting upon parts not concerned in the support of the uterus, and even tending to increase the obstacles in the way of its reposition, is not based upon physiological grounds, and must prove valueless, unless perhaps in certain cases of *procidentia*, where the mere retention of the organ within the pelvic cavity may be the prime object in view.

Permit me to advert to other methods, not new indeed as a class, but open to as serious objection as that above alluded to. The uterine neck, when in its normal condition, is not endowed with great sensitiveness, nor are the parts contiguous thus sensitive. But from whatever cause, let congestion and inflammation arise, or let nervous irritations occur, upon the contact of parts not designed to exist, and speedily another condition is present—one of hyperæsthesia—which is to be regarded most religiously in the treatment proposed or undertaken. Imagine, then, a uterus prolapsed for months, perhaps for years, congested or inflamed, every motion of the body carrying with it a local ache or a twinge in parts remote by reason of the extreme sympathies awakened with the diseased and distressed organ, and conceive again of enclosing such a cervix in an unyielding socket of wood or rubber or other possible material, or, indeed, of allowing it to rest in any way upon such an instrument, supported from without, for the implied and expressed purpose of maintaining the uterus in an elevated position, thus to secure *relief from suffering!* It need hardly be written, that such a support will, in the vast majority of cases, fail signally in accomplishing its object, where it does not indeed aggravate the symptoms, because of its unfortunate substitution of a still more unyielding and more irritating resting place for that to which the displaced organ has been already reduced. No wonder is it that the pessaries of years ago were confessedly pitiable failures, or that the profession are loth to acknowledge that any good can come of an instrument bearing the name. Beside the demerit suggested, great objection applies to a method which needs for its fulfillment external appliances which, in the constant motion of the wearer, must always remind her of her ailment, inducing by their sensible presence a condition of mind and body neither comfortable nor healthful. One at all conversant with the delicate sensitiveness of the female mind will look far and wide for means which will meet as well the mental as the physical acquirements of her case.

The same objection applies to all "abdominal pads" and "supporters," which are moreover entirely unphilosophical, tending, I believe, *always* to aggravate the cause of suffering, under the dangerous guise of temporary relief. Dr. Meigs is undoubtedly right in considering the "abdominal cavity" a *plenum*, making it subservient to the laws of hydrostatics, in that pressure externally applied is communicated in exact ratio to its walls inferiorly, superiorly and laterally. It matters not if that pressure be "directed upward" to relieve the womb of the weight of the bowels, it only tends to force the abdominal contents into the pelvic cavity, depressing rather than conducing to the elevation of the uterus.

Enough has been said to afford the premises for a brief exposition of a plan of treatment for this affection, differing in theory and practice from those advanced by your contributors, and advocated by men whose obstetric writings have deservedly secured for them a world-wide reputation.

I have endeavored, indirectly, to suggest the advantages to be sought for, as well as the disadvantages to be avoided, in the selection of an uterine support. First, it must be contrived in accordance with a just appreciation of the relationship of the parts concerned. It must be *philosophical*. Second, it must not involve such an unnatural interference with those relations, as, if unsuccessful, to prevent other means being used, having the same object in view. Third, it must elevate without pressing upon irritated or inflamed parts. It should be painless. Fourth, it must enhance the mental comfort and well-being of the patient, by being an ever present assistant, without being a constant reminder, and require the least possible interference from physician or patient. Fifth, it should not interfere with the integrity and complete performance of every function, marital or otherwise. Lastly, it should aim at completeness and permanency of cure in the least possible time.

I know of no uterine support which so fully meets these indications as the pessary of Dr. Hodge, of Philadelphia. It is eminently philosophical, addressing itself entirely to the relief of the parts, whose office it is to afford a perfect and elastic support to the uterus, and finishing its work when, by long rest and judicious general treatment, they have been enabled to regain a natural tension and capacity. It involves no change in the vaginal tissues, or such topical derangements of parts as would prevent its own disuse, and the subsequent trial of other means. I speak with the confidence of experience, in claiming for it also the power of elevating and suspending the displaced organ in such a manner as th t its cervix, never intended to be its support, and diseased because it has been abused in that capacity, is freed from contact with unyielding tissues, and from the weight of superincumbent organs. And this it does, when properly applied, with the alone temporary inconvenience of introduction. It requires no assistance from without, by straps or abdominal pads, and is worn *unconsciously* by the patient for weeks and months, with only care enough on her part to secure cleanliness of person. It interferes in no wise with the use of the syringe, the freedom of exit of menstrual fluids, or with the exercise of marital rights. It aims at, and secures in the majority of instances, a condition of mind and body freed from deleterious influences, and now able react most healthfully in all its parts, particularly in those whose duty it is to support the uterus, so that, at a variable period, the patient may be encouraged to hope for a more natural and complete freedom of power and function.

It has obviously been my purpose in this communication to avoid any incursion upon the wide field of controversy which separates the disciples of Dr. Bennett and Dr. Hodge, as the representative men of our day, so far as concerns uterine affections. I have simply presumed that there were some, who, searching after truth and effective means, had failed either to meet with Dr. Hodge's instrument, or to give it the thorough and tentative examination which secures a full appreciation of its merit. Its claims are so fully and ably set forth in the inventor's work on the Diseases of Women, that I shall have amply repaid myself if I induce any physician to study, still more, to test in practice, its admirable precepts.

As an apology, I may add, that in no other department o. practice have I found nearly so favorable results, in so large a proportion of cases, to attend the use of any single *curative* means, and I hope, at some future time, to give the profession, at length, the details of cases which have furnished my own mind with the most positive and conclusive proofs of the truthfulness of the statements made at present.

H. WEBSTER JONES, M. D.

---

*Note.*—Since the above was written I have learned that several applications have been recently made, to an eminent medical man of this city, for *cauterizing* irons, with which to "*cure* (!) prolapsus uteri." *Verb. sap. sat.*

